# Validity of DEXA-Derived Thigh Muscle Quantification Against AI-Assisted CT: Inter-Limb Asymmetry Provides Superior Agreement over Absolute Values

**DOI:** 10.3390/jcm15020594

**Published:** 2026-01-12

**Authors:** Do Kyung Lee

**Affiliations:** Department of Orthopedic Surgery, Samsung Changwon Hospital, Sungkyunkwan University School of Medicine, Changwon 51353, Republic of Korea; dr-lee331@hanmail.net

**Keywords:** knee osteoarthritis, sarcopenia, muscle asymmetry, quadriceps muscle, DEXA thigh segmentation, intramuscular adipose tissue (IMAT)

## Abstract

**Background/Objectives:** This study evaluated the validity of DEXA-derived muscle quantification by assessing its agreement with AI-assisted CT measurements of muscle volume and intramuscular adipose tissue. It also examined whether inter-limb asymmetry improves DEXA–CT agreement beyond absolute DEXA values. The influence of lower-limb rotation on DEXA measurements was assessed, and the study aimed to clarify how DEXA should be obtained and interpreted to more accurately reflect true muscle status. **Methods:** Fifty-two patients who completed CT and DEXA within 14 days were included. CT was used to obtain pure muscle volume and intramuscular adipose tissue (IMAT) using a standardized AI segmentation protocol, and corresponding DEXA thigh segmentation provided lean mass and fat percentage. Position-specific correlation analysis, regression, and Bland–Altman agreement testing were performed for 104 limbs. The same analyses were applied to inter-limb differences to isolate within-person asymmetry and reduce between-person variance. **Results:** DEXA lean mass correlated with CT pure muscle volume (r = 0.776, *p* < 0.001), and inter-limb asymmetry further improved alignment with CT (r = 0.857, *p* < 0.001). However, DEXA fat mass asymmetry demonstrated no association with CT IMAT asymmetry (r = −0.004, *p* = 0.979). When results were stratified by the recorded rotational groups, the highest correlation was observed in the neutral position (r = 0.900, *p* < 0.001). Bland–Altman analyses showed wide limits of agreement for all absolute measurements, whereas inter-limb asymmetry demonstrated markedly narrower limits of agreement, indicating superior numerical consistency. **Conclusions:** Absolute DEXA estimates showed limited agreement with CT and varied with limb position. Inter-limb asymmetry improved lean mass assessment, whereas fat mass and percentage did not correspond to CT-based IMAT. DEXA may therefore be used as a complementary tool for evaluating regional muscle quantity, but not for assessing muscle quality.

## 1. Introduction

Quadriceps atrophy is a well-recognized contributor to functional decline in patients with knee osteoarthritis (OA). Although age-related sarcopenia is involved, recent evidence indicates that pain accelerates asymmetric deterioration of quadriceps and gluteus maximus muscle volume and quality, accompanied by persistent fatty degeneration. These alterations represent a form of secondary, pain-related asymmetric sarcopenia. Mechanistic investigations further suggest that muscle atrophy in OA is not solely the result of disuse but is associated with inflammation-mediated disturbances in neuromuscular innervation [1,2,3]. Such asymmetric muscle impairment has been linked to patellofemoral pain syndrome, increased fall risk, and marked limb function deficit [1,2,4,5]. Increasing recognition of structure–symptom discordance [6,7] has shifted attention from radiologic disease to muscular factors that more directly influence pain and functional performance. Consistent with this concept, clinical trials have shown that muscle-strengthening exercise improves pain and function in OA [8,9,10], reinforcing the relevance of identifying and quantifying muscle loss and fatty degeneration as part of contemporary OA management.

A wide array of imaging and functional techniques has been adopted to quantify muscle volume and quality, including MRI, CT-based segmentation, and isokinetic dynamometry [11,12,13]. MRI and CT provide detailed volumetric assessment, yet their use in routine clinical practice is limited by cost, scanning burden, radiation concerns, and processing requirements. DEXA may serve as an accessible complementary tool for evaluating muscle status, given the practical limitations of CT and MRI, because it provides regional soft-tissue composition at low cost and without the need for post-processing. This enables efficient monitoring of muscle quantity and allows clinicians to deliver immediate feedback during routine outpatient care [13,14,15,16,17]. For assessing asymmetric quadriceps atrophy, which is closely associated with pain and functional decline in knee osteoarthritis [1], DEXA thigh segmentation involves manually defining a region of interest over the thigh and deriving lean and fat estimates from two-dimensional attenuation projections. However, DEXA cannot anatomically isolate individual muscles or separate intramuscular adipose tissue from subcutaneous or intermuscular fat, making its validity for thigh muscle quantification uncertain.

Prior studies have reported moderate to strong correlations between DEXA-derived thigh lean mass and muscle volume quantified by CT or MRI [11,18,19,20]; however, errors attributable to the two-dimensional projection nature of DEXA cannot be excluded. Unlike CT, which separates muscle and adipose tissue at the voxel level [1,21,22], DEXA integrates attenuation signals from skin, subcutaneous fat, fascia, and deep structures into a single projected pixel value, introducing numerical bias and limiting the accuracy of intramuscular adipose quantification. Previous investigations also indicate that DEXA measurements can be influenced by body size, hydration status, and systemic factors [23,24]. Importantly, lower-limb rotation may alter soft-tissue attenuation patterns, yet the positional dependence of DEXA thigh measurements has not been systematically evaluated [25]. These factors raise fundamental uncertainty regarding whether changes in DEXA-derived estimates reflect true physiologic variation in muscle volume or merely measurement noise, particularly when examinations are performed longitudinally [16,26,27]. Such ambiguity complicates the interpretation of serial trends and the communication of these findings to patients, and few prior studies have provided guidance on how DEXA-based changes should be interpreted in the context of repeated muscle monitoring [25].

Evaluating inter-limb asymmetry may mitigate these limitations by focusing on within-person left–right differences, thereby reducing the influence of individual-level factors that confound absolute measurements. This rationale is supported by observations of pain-related secondary sarcopenia, in which selective asymmetric atrophy commonly develops in the more painful limb [1,28,29], and aligns with indices such as the limb symmetry index (LSI), widely used in sports medicine to characterize functional imbalance and monitor recovery [30,31,32]. Similar principles are also applied in orthopedic practice, such as the use of the ankle–brachial index (ABI) to assess ischemic status through side-to-side comparison [33,34]. These conceptual parallels led to the hypothesis that asymmetry-based assessment could enhance the interpretability of DEXA-derived muscle measurements.

Accordingly, this study was designed to evaluate the validity of DEXA-derived muscle quantification by assessing its agreement with AI-assisted CT measurements of pure muscle volume and intramuscular adipose tissue (IMAT), to determine the extent to which lower-limb rotation affects DEXA measurements, and to examine whether inter-limb asymmetry provides superior DEXA–CT agreement compared with absolute DEXA values. The study further aimed to clarify how DEXA should be obtained and interpreted to more accurately reflect true muscle status during clinical evaluation.

## 2. Materials and Methods

### 2.1. Study Population and Design

This cohort study was designed as a retrospective cohort analysis to evaluate how accurately DEXA reflects thigh muscle quantity and quality compared with CT-based measurements obtained using an AI-assisted segmentation program, and to determine how DEXA results should be interpreted in patients with knee osteoarthritis. Patients scheduled to undergo unilateral primary total knee arthroplasty (TKA) between June 2021 and May 2024 were screened, and a total of 94 candidates were identified. The study was conducted in accordance with the Declaration of Helsinki and was approved by the Institutional Review Board of our institution (IRB No. SCMC 2024-07-004).

**Figure 1:**
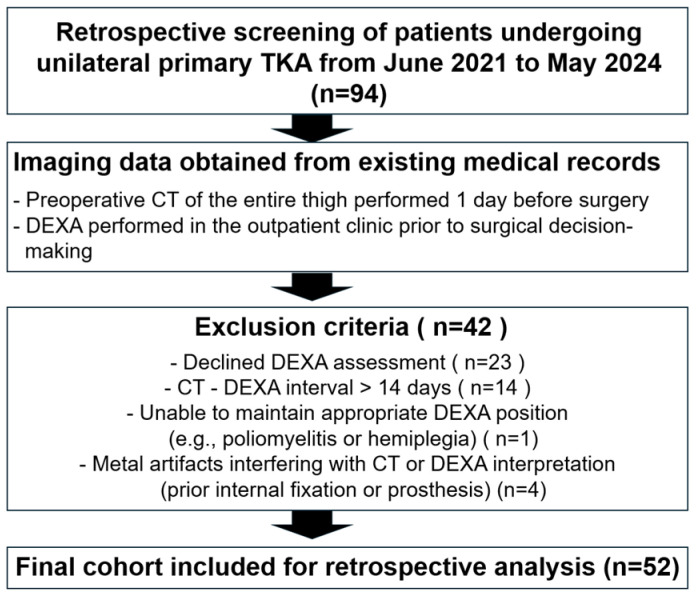
Flowchart showing patient enrollment and exclusion.

All patients underwent preoperative CT of the entire thigh one day before surgery. DEXA (Horizon W, S/N 302409M; Hologic Inc., Marlborough, MA, USA; software version 13.6.0.5) was performed at the outpatient clinic before surgical decision-making. Patients who declined DEXA assessment (n = 23) were excluded. Those with an interval exceeding 14 days between CT and DEXA (n = 14) were excluded to avoid potential mismatch caused by interval changes in muscle volume. Additional exclusions included patients unable to maintain an appropriate DEXA position due to conditions such as poliomyelitis or hemiplegia (n = 1), and patients with metal artifacts interfering with CT or DEXA interpretation, including prior internal fixation or prosthetic implants (n = 4). After application of these criteria, a final cohort of 52 patients who completed both CT and DEXA within a 2-week interval was included for analysis. The study flowchart is presented in Figure 1.

### 2.2. Patient Evaluation

#### 2.2.1. AI-Based Quantification of Muscle Volume and Intramuscular Adipose Tissue (IMAT) (Figure 2)

Preoperative thigh muscle volume and IMAT were quantified from CT scans using a standardized AI-assisted segmentation protocol [1].

**Figure 2:**
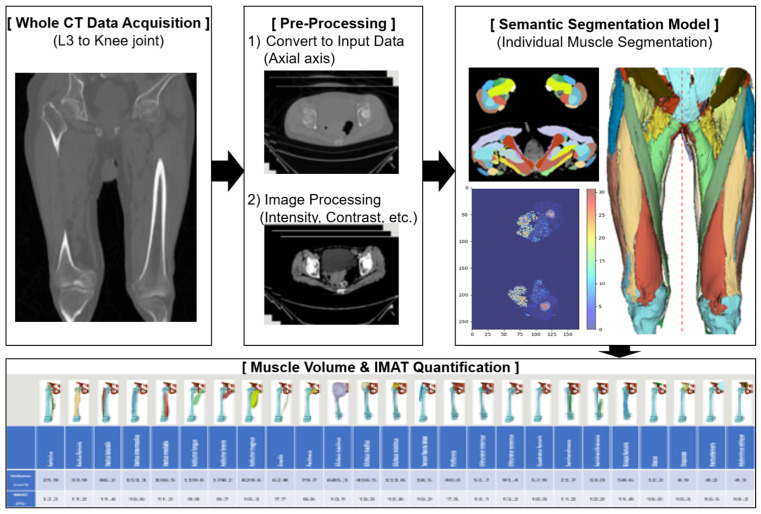
AI-based quantification of muscle volume and intramuscular adipose tissue (IMAT).

##### Semantic Segmentation Framework

A 3D semantic segmentation approach was applied to classify each voxel into muscle, adipose tissue, or bone, allowing for precise anatomical separation of individual thigh muscles. This voxel-wise method enables reproducible volumetric measurements by delineating complex boundaries and minimizing contamination from adjacent structures.

##### Segmentation Targets

The segmentation covered the full length of the thigh, beginning at the hip joint and extending distally to the knee joint. Within this region, the major thigh muscles were identified and labeled, including the sartorius, rectus femoris, vastus lateralis, vastus intermedius, vastus medialis, adductor longus, adductor brevis, adductor magnus, gracilis, semitendinosus, semimembranosus, and biceps femoris. Muscles with incomplete coverage due to variable starting levels (e.g., iliacus, psoas, iliopsoas, abdominal oblique, rectus abdominis, multifidus) or hip muscles outside the target region (e.g., gluteus medius/minimus/maximus, tensor fasciae latae, piriformis, obturator muscles, quadratus femoris) were excluded. All labels were reviewed jointly by radiology and anatomy specialists to ensure annotation accuracy.

##### Segmentation Model and Image Pre-Processing

Segmentation was performed using a UNETR-based architecture optimized for 3D medical imaging [1,21]. The model incorporates transformer-based encoding to capture global context and a U-shaped decoder to retain fine spatial detail.

CT images were pre-processed through the following measures:

- Intensity normalization to −57 to 164 HU;
- Gamma correction (γ = 2) to enhance muscle contrast;
- Foreground cropping to remove irrelevant regions.

The model achieved a Dice similarity coefficient of 0.91, indicating excellent agreement with expert-derived ground truth [35].

##### Extraction of Muscle Volume and IMAT

Each voxel was classified as skeletal muscle or intramuscular adipose tissue using deep learning-based semantic segmentation and HU thresholds, and subcutaneous or intermuscular fat compartments were excluded from all measurements. Pure muscle volume represented the sum of voxels identified as muscle only, whereas IMAT volume represented voxels classified as intramuscular fat.

Muscle and adipose volumes were derived by summing voxel volumes within their respective segmented regions. IMAT was identified using a validated HU threshold (−190 to −30 HU), and IMAT percentage was calculated as adipose volume divided by the corresponding muscle volume. Measurements were obtained separately for each limb to enable side-specific analysis.

#### 2.2.2. DEXA Measurement and Patient Grouping

##### Thigh Segmentation and DEXA Acquisition

All DEXA examinations were performed using a Hologic fan-beam DXA scanner (Horizon W, S/N307470M, Hologic Inc., Bedford, MA, USA). Image acquisition followed previously published lower-limb positioning protocols [24,36,37]. To evaluate how accurately DEXA reflected true thigh muscle volume, the right and left thighs were segmented separately, as illustrated in Figure 2. Segmentation levels were determined with reference to established methods [16,26,36], and every effort was made to ensure that only thigh musculature was included.

**Figure 3:**
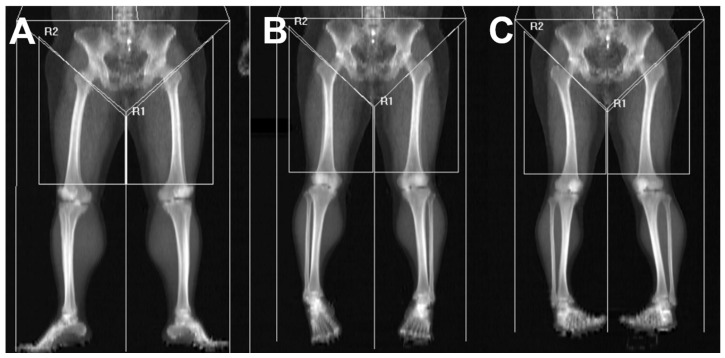
**Thigh muscle segmentation and subgrouping based on lower-limb rotation**. Lower-limb rotation was categorized according to the radiographic relationship between the tibia and fibula on DEXA scout imaging: (**A**) **External rotation** was defined when the fibula was largely obscured by the tibia, resulting in marked overlap. (**B**) **Neutral position** was defined when the interosseous space between the tibia and fibula was clearly visualized with appropriate width. (**C**) **Internal rotation** was defined when the interosseous space appeared excessively widened, indicating anterior displacement of the fibula relative to the tibia. R1 indicates left thigh segmentation, and R2 indicates right thigh segmentation on DEXA imaging.

The superior boundary was set at the intertrochanteric line to avoid inclusion of pelvic viscera or fluid-containing compartments, which cannot be reliably differentiated from muscle on DEXA [24]. The inferior boundary was drawn at the level of the patellar upper pole. A vertical center line originating from the symphysis pubis was used as the medial reference, and a parallel vertical line was placed laterally while ensuring that subcutaneous tissue was excluded and the entire thigh musculature remained within the region of interest. Despite adherence to standardized scanning protocols, visual review of DEXA images revealed variability in lower-limb rotation across participants. Because tibial–fibular overlap may influence DEXA-derived values, each scan was categorized into one of three subgroups—external rotation, neutral position, or internal rotation—based on the degree of overlap between the tibia and fibula. The rotation category was determined by an orthopedic surgeon with more than 10 years of specialized experience in knee surgery at a tertiary referral hospital. These groups were used for subsequent analyses (Figure 3). In addition, several patients demonstrated mismatched rotational alignment between the right and left lower limbs (e.g., one limb in external rotation and the contralateral limb in neutral or internal rotation). To account for this discrepancy and prevent misclassification bias, a separate “Mixed” group was created. This group included patients whose bilateral limb rotation on DEXA did not fall within the same positional category.

### 2.3. Outcome Measurement

Thigh muscle composition was quantified using AI-derived values from CT. For each muscle, intramuscular adipose tissue (IMAT) volume was calculated using the AI-generated IMAT percentage, and the sum of all individual muscle volumes was used to obtain total thigh muscle volume. The sum of each muscle’s IMAT volume yielded total thigh IMAT volume. Pure muscle volume was defined as thigh muscle volume minus thigh IMAT volume, and IMAT percentage was calculated as IMAT volume divided by thigh muscle volume. All measurements were obtained separately for the right and left limbs. From DEXA, lean mass, fat mass, and fat percentage were extracted for each thigh based on the predefined segmentation region.

### 2.4. Statistical Analysis

All CT-derived and DEXA-derived measurements were paired at the limb level, yielding 104 matched observations for absolute analyses and 52 paired observations for inter-limb difference analyses. Four analytic conditions were evaluated: the entire cohort as well as three positional subgroups (external rotation, neutral, internal rotation). Because several patients exhibited differing rotational alignment between limbs, a fourth rotational category (“Mixed”) was added for inter-limb analyses. The Mixed group consisted of cases in which right and left limbs belonged to different rotational categories. All statistical analyses were conducted using Python (version 3.9.13; Python Software Foundation, Wilmington, DE, USA) with the following libraries: NumPy (version 1.23.5), Pandas (version 1.5.3), SciPy (version 1.10.1), and Matplotlib (version 3.7.1). A two-sided *p*-value < 0.05 was considered statistically significant.

#### 2.4.1. Correlation and Regression Analyses

Pearson correlation coefficients were calculated to assess linear associations between DEXA-based metrics (lean mass, fat mass, and fat percentage) and CT-derived reference measures (pure muscle volume, IMAT volume, and IMAT percentage). Simple linear regression was performed using CT metrics as predictors and DEXA metrics as dependent variables. Regression intercepts and slopes were compared across rotational subgroups to examine the influence of lower-limb position on measurement linearity. Model fit was summarized using R^2^ values.

Inter-limb differences (right–left) were calculated for all CT-derived and DEXA-derived variables in 52 patients, and the same correlation and regression procedures were applied to determine whether asymmetry-based metrics demonstrated stronger associations than absolute values.

#### 2.4.2. Bland–Altman Agreement Analysis

Because correlation does not assess numerical agreement, Bland–Altman analysis was performed as the primary validation tool to evaluate whether DEXA variables can serve as surrogate markers for CT-based pure muscle volume and IMAT. CT-derived measurements were treated as the reference standard for evaluating numerical agreement. For each comparison (CT pure muscle volume vs. DEXA lean mass, CT IMAT percentage vs. DEXA fat percentage), the following indices were computed:

- Mean difference (bias);
- 95% limits of agreement (LoA) calculated as bias ± 1.96 × SD of the differences;
- Visual inspection for proportional bias across the measurement range.

The same Bland–Altman procedures were repeated for inter-limb differences (right–left) to determine whether asymmetry-based interpretation improved agreement compared with absolute measurements. Inter-limb differences were analyzed to reduce individual-level physiological variability and to isolate true asymmetry-related changes.

### 2.5. AI-Assisted Writing Disclosure

CT-based muscle volume and IMAT measurements were obtained using an AI-assisted segmentation tool following the protocol described above. An artificial intelligence language model (ChatGPT, GPT-4 series, OpenAI, San Francisco, CA, USA) was used to assist with English grammar refinement and sentence organization during manuscript preparation. The AI tool was not used for data analysis, statistical processing, image generation, or interpretation of study results.

## 3. Results

### 3.1. Agreement Between DEXA and CT Muscle Metrics

DEXA-derived lean mass demonstrated strong correlations with CT-pure muscle volume across the entire cohort (r = 0.776, both *p* < 0.001). Correlations between DEXA fat-related metrics and CT-derived IMAT were weaker (fat mass vs. IMAT volume: r = 0.513; fat percentage vs. IMAT percentage: r = 0.582; both *p* < 0.001). These patterns were consistent across limb positions and are summarized in Table 1.

### 3.2. Influence of Lower-Limb Rotation on DEXA–CT Relationships

**Figure 4:**
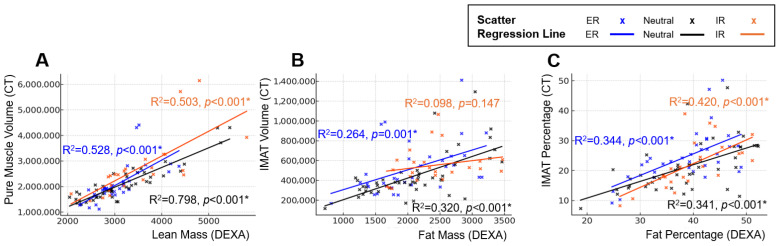
**Position-dependent regression characteristics between DEXA-derived body composition metrics and CT-based reference measurements.** Panels illustrate changes in regression slopes and model fit across external rotation, neutral, and internal rotation positions. Scatter plots and simple linear regression lines are shown for each position (External rotation = blue, Neutral = black, Internal rotation = orange): (**A**) Linear regression between DEXA lean mass and CT pure muscle volume. (**B**) Linear regression between DEXA fat mass and CT IMAT volume. (**C**) Linear regression between DEXA fat percentage and CT IMAT percentage. Across all analyses, regression slopes and explanatory power varied according to limb position, demonstrating the positional sensitivity of DEXA measurements compared with CT-based reference standards. An asterisk (*) indicates a statistically significant difference (*p* < 0.05).

Lower-extremity rotation altered the strength of association between DEXA and CT. The strongest correlations between DEXA-derived lean mass and CT-based muscle metrics were observed in the neutral position, followed by ER, with IR demonstrating the weakest association, indicating variable positional susceptibility. Fat-related metrics on DEXA showed a correlation with CT-derived IMAT volume and percentage; however, the correlation coefficients were lower than those between DEXA lean mass and CT pure muscle volume. This indicates a weaker overall correspondence between DEXA-derived fat measurements and CT-based quantification of IMAT. Position-specific regression slopes and R^2^ values similarly demonstrated rotation-dependent variability, supporting the positional sensitivity of DEXA absolute measurements (Figure 4).

### 3.3. Inter-Limb Differences Provide Stronger DEXA–CT Agreement

**Figure 5:**
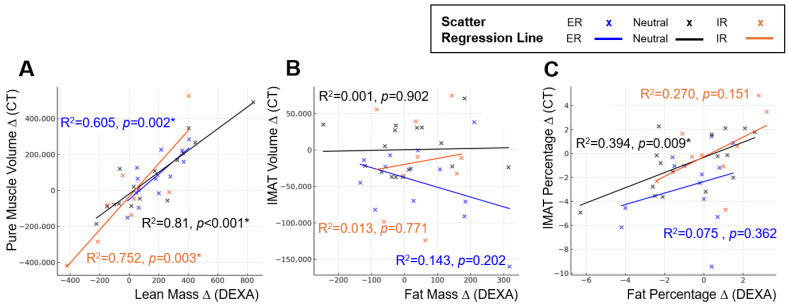
**Position-dependent regression characteristics between inter-limb differences in DEXA-derived body composition metrics and CT-based reference measurements.** Panels illustrate changes in regression slopes and model fit across external rotation, neutral, and internal rotation positions. Scatter plots and simple linear regression lines are shown for each position (external rotation = blue, neutral = black, internal rotation = orange): (**A**) Linear regression between inter-limb differences in DEXA lean mass and CT pure muscle volume. (**B**) Linear regression between inter-limb differences in DEXA fat mass and CT IMAT volume. (**C**) Linear regression between inter-limb differences in DEXA fat percentage and CT IMAT percentage. Across all comparisons, regression slopes and explanatory power differed by limb position, demonstrating the positional sensitivity of inter-limb DEXA measurements relative to CT-based standards. An asterisk (*) indicates a statistically significant difference (*p* < 0.05).

Side-to-side differences markedly improved agreement for muscle-related measures. DEXA lean mass difference showed stronger correlations with CT pure-muscle difference (r = 0.857) than absolute metrics, with reduced positional variability (Table 2). Differences in fat mass showed no meaningful association with differences in IMAT volume. Fat-percentage differences demonstrated a correlation with IMAT percentage differences only in the neutral position; however, the correlation coefficients were lower than those observed between DEXA lean mass differences and CT pure muscle volume differences, and no correlation was observed in the ER or IR positions. Position-specific regression slopes and R^2^ values are summarized in Figure 5. Taken together, the benefits of asymmetry analysis were specific to lean mass estimation. Fat mass and IMAT-related asymmetry did not demonstrate meaningful alignment with CT.

### 3.4. Bland–Altman Analysis: Absolute Measurements Exhibit Wide Disagreement

Bland–Altman analysis revealed substantial numerical disagreement between absolute DEXA and CT values (bias −1.75 × 10^6^; LoA −3.28 × 10^6^ to −0.22 × 10^6^). For CT pure muscle volume vs. DEXA lean mass, a large negative bias with very wide limits of agreement (LoA) indicated that DEXA consistently underestimated CT-derived muscle quantity with high variability across the measurement range. Similarly, DEXA fat percentage showed broad LoA when compared with CT IMAT percentage (bias +16.4%; LoA +2.3% to +30.6%), demonstrating limited reliability for assessing absolute adipose infiltration.

### 3.5. Bland–Altman Analysis: Inter-Limb Differences Improve Numerical Agreement

Inter-limb differences produced markedly narrower LoA for both lean mass and fat-percentage comparisons.

**Figure 6:**
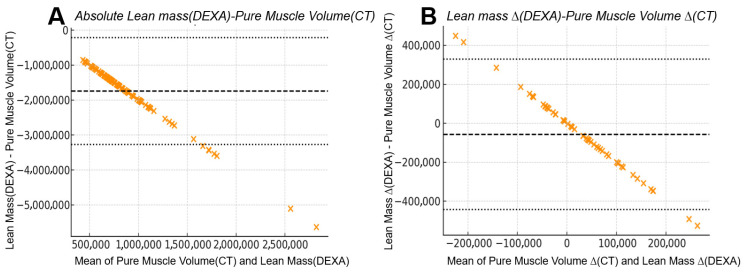
**Bland–Altman comparison between CT-derived pure muscle volume and DEXA-derived lean mass:** (**A**) Absolute measurements from 104 limbs showed a mean bias of −1.75 × 10^6^ units with wide 95% limits of agreement from −3.28 × 10^6^ to −0.22 × 10^6^, indicating substantial numerical disagreement between the two modalities despite their strong linear correlation. (**B**) Inter-limb differences (right–left) from 52 patients demonstrated markedly improved agreement, with the mean bias reduced to −5.76 × 10^4^ units and narrower 95% limits of agreement (−4.43 × 10^5^ to +3.28 × 10^5^). The narrower limits of agreement in inter-limb differences reflect a reduced influence of systemic components that affect absolute DEXA measurements, resulting in improved correspondence with CT-derived asymmetry after subtraction.

**Figure 7:**
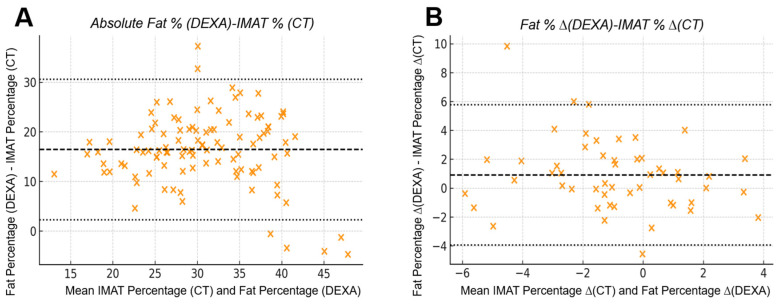
**Bland–Altman agreement between CT-derived IMAT percentage and DEXA fat percentage:** (**A**) Absolute analysis demonstrated limited agreement, with a mean bias of +0.91% and wide limits of agreement (−3.96% to +5.78%), indicating substantial numerical variability between modalities when quantifying absolute intramuscular adiposity. (**B**) Inter-limb differences showed improved consistency, with narrower scatter and the same bias (+0.91%) and limits of agreement (−3.96% to +5.78%) when evaluating asymmetry. The improved consistency in inter-limb differences reflects a reduced influence of systemic factors that affect absolute DEXA values, resulting in closer correspondence with CT-derived asymmetry when right–left subtraction is applied.

The bias between DEXA lean mass difference and CT pure muscle difference was close to zero, and LoA intervals were substantially reduced compared with absolute values (bias −5.76 × 10^4^; LoA −4.43 × 10^5^ to +3.28 × 10^5^). DEXA fat percentage difference also showed improved alignment with CT IMAT percentage difference (bias +0.91%; LoA −3.96% to +5.78%). Inter-limb differences showed improved numerical agreement between DEXA and CT, with narrower limits of agreement and reduced scatter compared with absolute measurements. This pattern reflects a closer correspondence between DEXA-derived asymmetry and CT-derived asymmetry (Figure 6 and Figure 7).

## 4. Discussion

**Figure 8:**
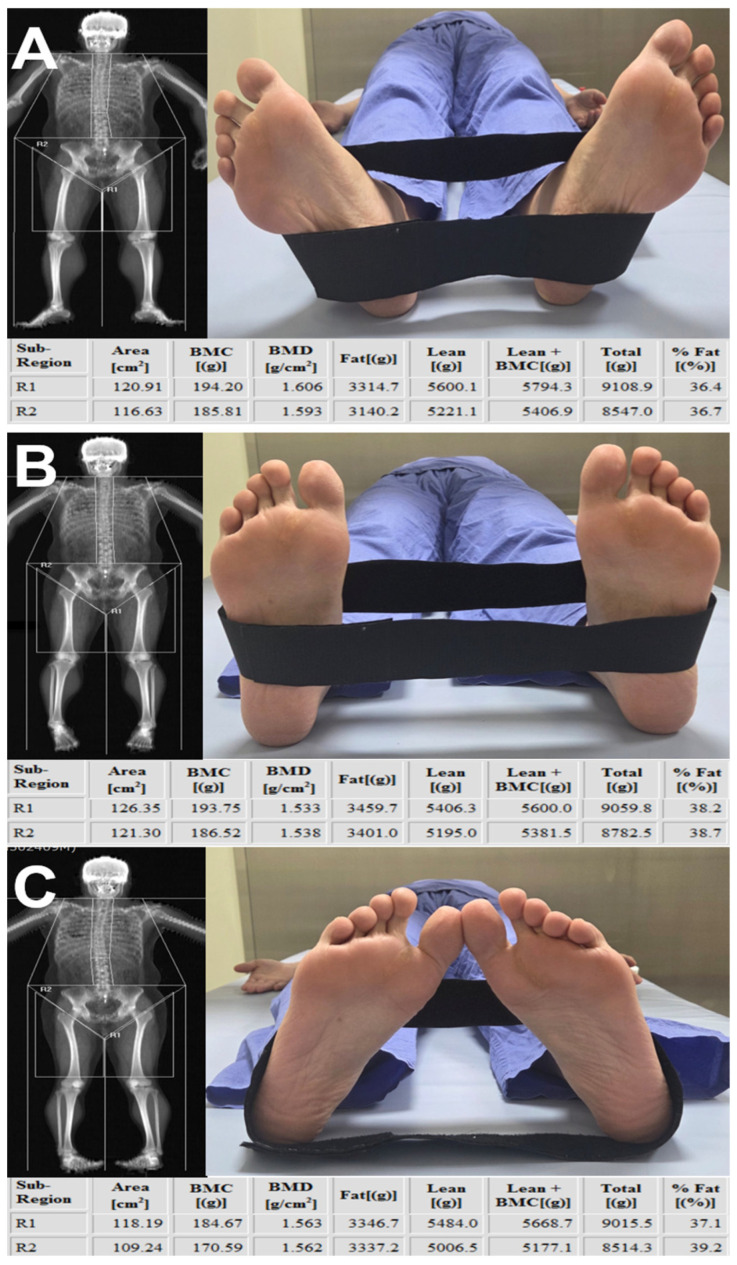
**Effect of lower-limb rotation on DEXA-derived measurements.** In the same patient, DEXA scans were obtained consecutively on the same day without an interval while the lower limb was positioned in (**A**) external rotation, (**B**) neutral alignment, and (**C**) internal rotation. The comparison demonstrates that DEXA-derived values within the segmented thigh region change substantially with limb rotation. R1 indicates left thigh segmentation, and R2 indicates right thigh segmentation on DEXA imaging.

This study found that although DEXA-derived lean mass and fat percentage showed moderate-to-strong linear associations with CT-based pure muscle volume and IMAT percentage, these relationships did not translate into reliable numerical agreement. Our results demonstrated that lower-limb rotation influences all DEXA-derived parameters. This effect appears to stem from positional alterations that modify the distribution of attenuation signals used for tissue classification (Figure 8). Thigh segmentation on DEXA requires manual identification of anatomical boundaries rather than a fully automated procedure, and variability in the placement of these boundaries during serial assessments represents an additional source of fluctuation [16,26,27]. Previous studies have also shown that hydration status, age, comorbid conditions, and body weight changes contribute to variability in lean mass estimation [23,24]. These multifactorial influences indicate that absolute DEXA values may fluctuate between examinations, limiting their stability for quantifying true changes in muscle volume. As demonstrated in our study, considerable variation in lower-limb rotation was observed during DEXA acquisition, despite the use of straps intended to standardize patient positioning. Although examiner-related differences in positioning technique may contribute to this variability, patient-related factors likely play a substantial role. Varus alignment or rotational deformities associated with osteoarthritis can make it difficult for patients to maintain a uniform posture, and some older individuals with muscle weakness or knee joint deformity may be unable to sustain the recommended slight internal rotation during scanning. These patient-related limitations suggest that absolute DEXA-derived lean-mass measurements have intrinsic constraints in reproducibly representing true muscle volume, leading to substantial measurement variability. This interpretation is supported by our Bland–Altman analysis, which demonstrated wide limits of agreement for absolute values.

In contrast, inter-limb asymmetry demonstrated higher correlation coefficients than absolute DEXA measurements and remained more robust across rotational variability. Bland–Altman analysis further showed markedly narrower limits of agreement with mean differences approaching zero. These findings suggest that inter-limb differences partially reduce systemic bias, including height, weight, BMI, hydration status, comorbid conditions, and variations in limb rotation during scanning, all of which can affect absolute DEXA values. Interestingly, even the Mixed group, which represented the least favorable condition because of inconsistent rotational alignment between limbs, demonstrated a high correlation for lean mass differences. This observation indicates that asymmetry-based assessment retains meaningful robustness even when positional variability occurs, likely because inter-limb subtraction reduces systemic and projection-related biases that disproportionately influence absolute measurements. As a result, applying inter-limb asymmetry yielded closer agreement with CT-based muscle measurements than absolute DEXA values, reflecting a measurable improvement in alignment between the two modalities. However, the benefits of asymmetry analysis were specific to lean mass estimation. Fat mass and IMAT-related asymmetry did not demonstrate meaningful alignment with CT, indicating that DEXA cannot reliably capture inter-limb differences in muscle quality. The correlation with CT-based asymmetry was strongest in the neutral and internally rotated positions, indicating that evaluating inter-limb asymmetry under standardized positioning conditions more accurately reflects true muscle status. Nevertheless, asymmetry did not eliminate positional dependence entirely. This highlights the importance of maintaining a consistent and standardized posture protocol during DEXA acquisition and the need to consider limb rotation when interpreting DEXA-derived measurements. In addition, the absence of external validation limits the generalizability of our findings, and further studies using independent validation cohorts will be necessary to confirm the robustness of asymmetry-based assessment.

Asymmetry-based metrics are well established in sports medicine as a standard approach for evaluating muscle status, particularly when determining readiness to return to sport after injury [30,31,32]. This concept is also relevant to knee osteoarthritis. Recent work shows that pain-related secondary sarcopenia is not a generalized decline in muscle mass but a pain-driven process that produces asymmetric muscle volume loss, particularly in the quadriceps and gluteus maximus of the more painful limb [1]. This biological predisposition toward asymmetry offers a compelling rationale for evaluating inter-limb differences when assessing muscle status in osteoarthritis. Within this context, DEXA-based inter-limb asymmetry provides a practical means for repeated monitoring, allowing clinicians to determine whether muscle status improves with strengthening interventions or whether persistent pain induces progressive atrophy, while avoiding the higher cost and radiation exposure of MRI and CT.

Although DEXA can support longitudinal muscle assessment, it cannot replace CT or MRI, which remain the reference modalities for detailed volumetric or compositional evaluation because they can anatomically differentiate muscle from adipose compartments [38,39,40]. CT and MRI require substantial post-processing for segmentation, which limits their suitability for point-of-care clinical decision making. DEXA, in contrast, offers rapid region-specific assessment with lower operator dependence than ultrasound or bioimpedance analysis, making it useful as a complementary tool in situations where repeated CT or MRI would be impractical. Even so, its sensitivity to limb positioning necessitates strict adherence to standardized scanning protocols, and clinicians must consider rotational variation when interpreting results. Under appropriate conditions, however, DEXA-based asymmetry can provide clinically meaningful information that complements advanced imaging and supports longitudinal monitoring in routine care.

However, EXA fat percentage demonstrated moderate correlation with CT-based IMAT percentage, and Bland–Altman analysis indicated considerable numerical disparity for absolute IMAT metrics. This limitation reflects the fundamental nature of DEXA, which quantifies total soft-tissue attenuation within a region of interest and cannot differentiate intramuscular fat from subcutaneous or intermuscular adipose components [13,23,24]. Accordingly, DEXA should be regarded primarily as an indirect modality for monitoring regional muscle quantity rather than a tool for detailed tissue characterization [40]. Inter-limb comparison provides improved reliability, but the method is not appropriate for precise evaluation of muscle quality or for tracking IMAT progression.

Several limitations should be acknowledged. The sample size was modest, and larger studies may reveal additional associations. Although CT-based muscle volume and IMAT serve as robust reference standards, imaging-derived metrics may not fully represent physiologic muscle function. Importantly, the two modalities differ fundamentally in their segmentation principles even when aligned using the same anatomical landmarks. CT provides voxel-level quantification of skeletal muscle and intramuscular adipose tissue, whereas DEXA is used to derive lean and fat estimates from two-dimensional soft-tissue projections. As a result, the analyzed regions cannot be assumed to be perfectly identical, and a partial mismatch between CT-derived muscle volume or IMAT and the DEXA thigh region of interest is unavoidable. This structural difference should be considered when interpreting cross-modal comparisons. Prior reports suggest that asymmetric sarcopenia extends into the hip musculature [1]; however, DEXA segmentation is inherently limited in this region due to overlapping visceral structures [13]. Future studies should determine whether DEXA-derived asymmetry correlates with functional outcomes, symptom burden, or postoperative recovery trajectories. Lastly, segmentation boundaries were manually placed according to anatomical landmarks; small variations in line placement between scans were unavoidable and were considered potential sources of absolute lean-mass variability.

Despite these limitations, the present study provides a practical framework for interpreting DEXA-derived thigh segmentation in knee osteoarthritis by combining AI-assisted CT quantification with a positional evaluation of DEXA performance. Absolute DEXA metrics lacked the numerical agreement and positional stability required for precise quantification of muscle volume or IMAT, whereas inter-limb asymmetry demonstrated greater robustness when examinations were conducted under standardized positioning. These findings indicate that asymmetry can serve as a supplemental marker of relative muscle imbalance rather than a stand-alone quantitative measure. Although CT and MRI remain the reference standards for detailed volumetric and qualitative assessment, their cost, radiation exposure, and substantial post-processing requirements limit their suitability for repeated outpatient monitoring. The segmentation and reconstruction processes required for advanced imaging further restrict their ability to provide real-time feedback to patients regarding muscle status. DEXA offers a distinct advantage in this regard because its results are available immediately at the point of care and can be used to monitor longitudinal changes in regional muscle quantity when interpreted within standardized acquisition protocols. Inter-limb asymmetry assessment with DEXA, therefore, provides a practical method for detecting and tracking muscle imbalance in routine clinical practice, complementing but not replacing more advanced imaging modalities.

## 5. Conclusions

Although absolute DEXA metrics correlated with CT-derived muscle measurements, their numerical agreement was limited and highly dependent on limb positioning. In contrast, inter-limb asymmetry demonstrated substantially stronger correlations and narrower limits of agreement than absolute values, particularly for thigh muscle lean mass assessment. Asymmetry-based interpretation, therefore, improves the reliability of DEXA-derived lean mass evaluation, but fat mass and IMAT measurements remain unreliable even when asymmetry is considered. Consequently, DEXA may serve as a complementary tool for assessing regional muscle quantity, whereas muscle quality, including IMAT volume and percentage, cannot be accurately evaluated using DEXA.

## Figures and Tables

**Table 1:** **Correlation between DEXA-derived measurements and CT-based muscle volume and IMAT, and the influence of lower-extremity rotation on the agreement between modalities.** The table presents Pearson correlation coefficients and *p*-values for the associations between DEXA lean mass, fat mass, and fat percentage and their corresponding CT-based reference metrics, including pure muscle volume, IMAT volume, and IMAT percentage. Correlation analyses were performed for each lower-extremity position (external rotation, neutral, and internal rotation) as well as for the overall cohort to evaluate position-dependent changes in measurement agreement. An asterisk (*) indicates a statistically significant difference (*p* < 0.05).

		ER (n = 34)	Neutral (n = 46)	IR (n = 24)	Total (n = 104)
Lean Mass (DEXA) –Pure Muscle Volume (CT)	r	0.715	0.905	0.720	0.776
	*p*-value	<0.001 *	<0.001 *	<0.001 *	<0.001 *
Fat Mass (DEXA) –IMAT Volume (CT)	r	0.464	0.636	0.234	0.513
	*p*-value	0.006 *	<0.001 *	0.27	<0.001 *
Fat Percentage (DEXA) –IMAT Percentage (CT)	r	0.587	0.584	0.648	0.582
	*p*-value	<0.001 *	<0.001 *	<0.001 *	<0.001 *

**Table 2:** **Correlation between inter-limb differences in DEXA-derived measurements and CT-based muscle and IMAT metrics across lower-extremity positions.** The table summarizes Pearson correlation coefficients and *p*-values for the associations between right–left differences in DEXA lean mass, fat mass, and fat percentage and corresponding CT-derived differences in pure muscle volume, IMAT volume, and IMAT percentage. Pure muscle volume differences consistently showed high correlations with minimal positional variation. An asterisk (*) indicates a statistically significant difference (*p* < 0.05).

		ER (n = 13)	Neutral (n = 16)	IR (n = 9)	Mixed (n = 14)	Total (n = 52)
Lean Mass Difference (DEXA) –Pure Muscle Volume Difference (CT)	r	0.778	0.900	0.867	0.863	0.857
	*p*-value	0.002 *	<0.001 *	0.003 *	<0.001 *	<0.001 *
Fat Mass Difference (DEXA) –IMAT Volume Difference (CT)	r	−0.378	0.034	0.114	0.304	−0.004
	*p*-value	0.202	0.902	0.771	0.290	0.979
Fat Percentage Difference (DEXA) –IMAT Percentage Difference (CT)	r	0.274	0.628	0.520	0.735	0.552
	*p*-value	0.362	0.009 *	0.151	0.003 *	<0.001 *

## Data Availability

The data underlying this study consist of clinical CT and DEXA imaging datasets stored in the institutional database of the Sungkyunkwan University Samsung Changwon Hospital. Due to ethical and legal restrictions related to patient privacy and institutional policy, these data are not publicly available. De-identified data may be obtained from the corresponding author upon reasonable request and with permission of the Institutional Review Board.
